# Immune microenvironment infiltration landscape and immune-related subtypes in prostate cancer

**DOI:** 10.3389/fimmu.2022.1001297

**Published:** 2023-01-09

**Authors:** Wei Wu, Xin’an Wang, Wei Le, Chang Lu, Haopeng Li, Yaru Zhu, Xi Chen, Wenbin An, Chengdang Xu, Qiang Wu, Licheng Wang

**Affiliations:** ^1^ Department of Urology, Tongji Hospital, School of Medicine, Tongji University, Shanghai, China; ^2^ Organ Transplantation Clinical Medical Center of Xiamen University, Department of Organ Transplantation, Xiang’an Hospital of Xiamen University, School of Medicine, Xiamen University, Xiamen, Fujian, China

**Keywords:** prostate cancer, immune cell infiltration, tumor microenvironment, immune checkpoint, tumor mutation burden

## Abstract

**Background:**

The tumor microenvironment (TME) primarily comprises cancer cells, cancer-infiltrating immune cells, and stromal cells. The tumor cells alter the TME by secreting signaling molecules to induce immune tolerance. The immune cell infiltrating the TME influences the prognosis of patients with cancers. However, immune cell infiltration (ICI) in the TME of patients with prostate cancer (PC) has not yet been studied.

**Methods:**

In this study, we used Cell-type Identification by Estimating Relative Subsets of RNA Transcripts (CIBERSORT) and Estimation of Stromal and Immune cells in Malignant Tumors using Expression data (ESTIMATE) algorithms to identify three ICI clusters based on 1,099 genes associated with ICI in the TME. The patients were classified into three distinct ICI gene clusters based on overlapping differentially expressed genes in ICI clusters. Furthermore, the ICI scores were calculated using principal component analysis.

**Results:**

The results revealed that patients with high ICI scores had poor prognoses and reduced expression of immune-checkpoint genes and immune-related genes. Furthermore, the transforming growth factor-beta (TGF-β) and WNT-β signaling pathways were enriched in the high ICI score subgroup, which suggests that suppression of T cells could contribute to poor prognosis of patients with PC. A positive correlation was observed between the high-ICI-score subgroup and the high tumor mutation burden (TMB) value. Patients with low ICI scores could benefit from immunotherapy, indicating that the ICI score could be used to predict the efficacy of immunotherapeutic response.

**Conclusions:**

In summary, we provide a comprehensive overview of the landscape of ICI in PC, which could aid in designing the strategies for immunotherapy for patients with PC.

## Introduction

Prostate cancer (PC) is the most common cancer and the second leading cause of cancer-related death in male patients (1). Despite advancements in our understanding of the pathogenesis and treatment of patients with PC (2–5), it continues to be a primary health concern. Approximately 1.3 million new PC cases were diagnosed worldwide in a year (6). Prostate-specific antigen (PSA) and Gleason scores based on the histomorphology of the tissue were recently updated and are still widely used to measure the aggressiveness of PC in clinical settings (7–9). The therapeutic strategies for PC include androgen deprivation therapy (ADT) and chemotherapy; however, patients may develop resistance to ADT and chemotherapy. Hence, there is an urgent need to develop novel therapeutic strategies to improve survival outcomes and ensure disease control.

Nowadays, multiple immune checkpoint inhibitors that block programmed cell death protein 1 (PD1) and cytotoxic T lymphocyte-associated antigen 4 (CTLA4) are used for the treatment of multiple cancers like melanoma, kidney and non-small-cell lung carcinomas, *etc*. (10). Therefore, immunotherapy could be a promising therapeutic strategy for the treatment of cancers. A study has demonstrated that the combined use of CTLA4 blockade and irradiated prostate cancer vaccines decreases the occurrence and severity of prostatic tumors in TRAMP mice. This indicates that the tissue-specific antigens in PC could be used as novel targets for immunotherapy (11). However, immunotherapy has limited benefits for patients with PC compared with “immunologically responsive” cancers (12). PCs were traditionally deemed as an immunologically “cold” cancer with low tumor mutation burden (TMB), complex tumor microenvironment (TME), and immunogenicity (13). The PC cells are protected by several immune-related mechanisms from the immune cells, such as the TGF-β pathway, regulatory T cells, and myeloid-derived suppressor cells (14). Overall, only a fraction of patients with PCs is susceptible to immunotherapy. However, screening of immunosensitive patients with PC is still a major concern. In prostate cancer, biochemical recurrence (BCR) was defined as a rise in the blood level of prostate-specific antigen to two consecutive measurements of 0.2 ng ml^−1^ or greater after treatment with surgery or radiation. Therefore, BCR-free survival is a unique and important prognostic biomarker for PC. In the past few decades, the advent of high-throughput screening techniques, like next-generation sequencing, has helped uncover biological information related to tumorigenesis, specifically PC (15, 16). In this study, we used Estimation of Stromal and Immune cells in Malignant Tumors using Expression data (ESTIMATE) and Cell-type Identification by Estimating Relative Subsets of RNA Transcripts (CIBERSORT) algorithms to analyze the transcriptome profiles of patients with PC and obtain comprehensive intrinsic–tumoral immune landscape (17, 18). Based on the infiltration patterns of 22 immune cells, stromal score, and immune score, the samples were divided into three distinct immune cell infiltration (ICI) subgroups. Next, the patients with PC were divided into three gene clusters based on overlapping differentially expressed genes (DEGs) in three ICI subgroups. Furthermore, the ICI score was calculated to understand the immune landscape of PC, which could be used to predict the immunotherapy response and prognosis of patients with PC. These results together reveal that ICI scores could be used to predict immunotherapy response and to design immunotherapeutic strategies for the treatment of patients with PC.

## Materials and methods

### Data collection and pre-processing

Transcriptomic data of 1,099 patients with PC were obtained from publicly available databases like the Cancer Genome Atlas-Prostate Adenocarcinoma (TCGA-PRAD), Gene Expression Omnibus (GEO; GSE70768, GSE70769, and GSE116918), and Chinese Prostate Cancer Genome and Epigenome Atlas (CPGEA). The RNA sequencing data [RNA-seq; fragments per kilobase million (FPKM)] from TCGA-PRAD was retrieved using the University of California Santa Cruz (UCSC) Xena browser (https://xenabrowser.net/datapages/). The microarray datasets like GSE116918, GSE70768, and GSE70769) were retrieved from the GEO database (https://www.ncbi.nlm.nih.gov/geo/). RNA-seq was retrieved from the CPGEA database (http://www.cpgea.com/). The transcript per million values of TCGA and CPGEA were converted to the FPKM values, which were identical to the microarray values (19). The “ComBat” algorithm in the R package was used to eliminate the batch effect of non-biological and technical biases from each of the five datasets (20). The clinical information of samples including age, Gleason score, T stage, N stage, *etc*., were collected. The somatic mutation data of TCGA-PRAD were retrieved from the UCSC Xena browser.

### Unsupervised clustering analysis of PC

CIBERSORT is an analytical tool that estimates 22 types of ICI in tumors *via* 500 permutations (18). The CIBERSORT algorithm in the R package was used to quantify the extent of ICI in PC tissues. The ESTIMATE algorithm was used to calculate the stromal and immune content (stromal score and immune score) in all patients with PC (17). Hierarchical agglomerative clustering of PC was performed based on the ICI patterns. The number of clusters and stability was determined using the “ConsensuClusterPlus” R package based on unsupervised clustering “Pam” method per Euclidean and Ward’s linkage (21) and 500 times repeat to confirm the clustering stability.

### Identification of DEGs associated with the ICI phenotype

The patients with PC were classified into different ICI clusters based on ICI in the tumor. Linear models for microarray data (limma) R package were used to identify the DEGs in ICI clusters, and the cutoff value was set as *p*<0.05 (adjusted) and |log fold-change| >1 (22). The hub genes were screened from DEGs using Cytoscape (3.8.2). The differential analysis of CNDP2 and SERPINH1 was processed on the UALCAN database (http://ualcan.path.uab.edu/), which was mainly based on the TCGA database. Additionally, the expression of hub genes in PC and normal tissue was validated using immunohistochemistry (IHC) from the Human Protein Atlas (HPA) database (23, 24). The antibodies used for IHC were CNDP2 (CAB026196), HSPA9 (CAB005219), KPNB1 (HPA029878), and SERPINH1 (CAB004441). Furthermore, the correlation between the expression of hub genes and ICI in the tumor was determined using Tumor Immune Estimation Resource (TIMER, https://cistrome.shinyapps.io/timer/, (25).

### Cell culture and real-time quantitative PCR for hub genes

Immortalized prostate cell lines like RWPE-1 and prostate cancer cell lines like DU145, PC-3, C4-2, and 22RV-1 were purchased from the Chinese Academy of Science Cell Bank (Shanghai, China). RWPE-1, DU145, PC-3, C4-2, and 22RV-1 cells were cultured in Roswell Park Memorial Institute-1640 medium (11875101, Gibco) supplemented with 10% fetal bovine serum (1009941, Gibco) and 1% streptomycin/penicillin (15140122, Gibco). All cells were maintained at 5% CO_2_ at 37°C. The cell lines were not contaminated with mycoplasma.

Total RNA was extracted from RWPE-1, DU145, PC-3, C4-2, and 22RV-1 using TRIzol (15596018, Thermo Fisher Scientific). The purity and the concentration of the extracted RNA were determined. Moreover, 500 μg RNA was reverse-transcribed to complementary DNA (cDNA) using PrimeScript TR Master Mix reagent (RR036A, TaKaRa). Subsequently, the reaction mixture was prepared using cDNA, primers (Sangon Biotech), and PowerUp SYBR Green (A25742, Thermo) to perform quantitative PCR (qPCR). The primers were designed using National Center for Biotechnology Information (https://www.ncbi.nlm.nih.gov/), and the primer sequence is listed in **Supplementary Table S1**. *GAPDH* was used as the internal control.

### Cell proliferation assay

In total, 1,000 cells/well were seeded in 96-well plates. After culturing the cells for a period of time, the cells were incubated with 3-(4, 5-dimethylthiazol-2-yl)-2,5-diphenyltetrazolium solution for 1 h. The growth of the cells was detected by measuring the OD value at 490-nm wavelength. The siRNA sequences are listed in **Supplementary Table S1**.

### Western blotting

LNCap cells were cultured in six-well plates and lysed in RIPA buffer with 1% phosphatase and protease inhibitors. Then, the cell protein contents were quantified *via* bicinchoninic acid protein quantification kits. The amount (∼50 μg) of proteins was separated and transferred onto NC membranes (Millipore). The blocking membranes used were NC for 1 h. The following primary antibodies were used: rabbit *CNDP2* (1:500, PROTEINTECH, catalog number: 14925-1-AP), rabbit *SERPINH1* (1:500, HUABIO, R1511-11), and rabbit GAPDH (1:500, Cell Signaling Technology, MA, USA) to incubate overnight at 4°C. On the next day, the membrane was washed three times (10 min/each time) with 1× TBST and incubated with horseradish peroxidase-conjugated secondary antibodies for 1 h at room temperature. The membranes were immersed in ECL exposure system (Thermo Fisher Scientific) to develop luminescence.

### Transwell invasion assay

Cell invasion was analyzed using Transwell chambers pre-coated with 20% Matrigel (Corning, NY, USA) for 18 h at 37°C. Then, 6 × 10^4^ LNCap cells in serum-free Minimal Essential Medium were added to the upper chambers, and 20% fetal bovine serum of Dulbecco’s Modified Eagle’s Medium was added to the lower chambers. After incubation for 18 h at 37°C, the non-invaded cells were clear, and invading cells went through into the lower chambers and were fixed in absolute ethanol for 10 min and stained in 0.5% crystal violet for 15 min at room temperature. The invaded cells were counted in one random field from each chamber using an inverted light microscope (Nikon).

### 5-Ethynyl-20-deoxyuridine assay

5-Ethynyl-20-deoxyuridine (EdU) incorporation assay was performed using by Clict-ITTM EdU (Thermo, C10420) according to the manufacturer’s instructions. The cells were fixed in 4% paraformaldehyde (Sigma, USA), briefly incubated with 20 uM EdU for 1 h, and incubated in PBS + 3%BSA. Then, click-iT reaction reagents were used to incubate the cells for 0.5 h. Blocking with 3% BSA/0.05% Tween-20/PBS (PBST block buffer) was carried out for 1 h at room temperature. The cells were incubated with a primary antibody—anti-Biotin (Rabbit, Abcam, ab53494)—diluted in blocking solution for 2 h overnight at 4°C, washed three times with PBST, incubated with secondary antibody—Anti-Rabbit IgG Alexa Fluor 647 (Invitrogen, A31573)—in the same solution for 1 h at RT, and washed three times with PBST. To the second wash, 0.1 μg/ml DAPI was added. The cells were observed using an inverted immunofluorescence microscope (Nikon) at ×40 magnification.

### Cell apoptosis analysis

Cell apoptosis was carried out using Annexin V-FITC Apoptosis Detection Kit (Bestbio, China). Pulmonary microvascular endothelial cells were collected in six-well plates with 2 × 10^5^ cells/ml and incubated at 4°C overnight. The cells were treated with different concentrations of Dex for 1 h prior to exposure to 15% IR serum for 24 h. After having been washed with PBS, the cells were suspended in 400 μl binding buffer at a concentration of 2 × 10^6^ cells/ml, after which the cells were incubated in 10 μl Annexin V-FITC for 15 min and 15 μl propidium iodide for 20 min at 4°C in the dark. The treated cells were analyzed by flow cytometry (Biosciences Inc., China).

### ICI score construction

Based on the unsupervised clustering of DEGs, the ICI score was calculated to construct the ICI model for all patients with PC. The patients with PC were divided into distinct ICI gene clusters based on overlapping DEGs. The Pearson correlation was used to analyze the relation between DEGs and clustering features. The gene expression values positively related with clusters were named as the ICI genetype A; the rest of the DEGs were called ICI genetype B, respectively. The Boruta algorithm R package was used to further reduce the dimensionality of the different ICI gene signatures (26). The principal component 1 was used to extract the principal component analysis (PCA) value as the signature score. The ICI score for each patient was calculated using the gene expression grade index (27).


$$ICI\;score=\;\sum^{}PC1A-\;\sum^{}PC1B$$

### Somatic gene mutation analysis

The corresponding somatic mutation data of patients from the TCGA-PRAD dataset was retrieved using the UCSC Xena browser (https://xenabrowser.net/datapages/). The TMB of the patients was calculated by the total number of non-synonymous mutations in PC. To identify the correlation between somatic gene mutation and the ICI score, the patients with PC were divided into low- and high-ICI-score subgroups using the “maftool” R package (28). The top 20 driver mutation genes were further analyzed.

### Transcriptomic data and clinical features of patients treated with immunotherapy

The Tumor Immune Dysfunction and Exclusion (TIDE, http://tide.dfci.harvard.edu/) database was used to explore immune infiltration and T cell dysfunction. The TIDE database was used to determine the status of T cells in patients with PC (29). The immunophenotype scores (IPS) were used to determine the prognostic value of the ICI score in predicting a patient’s response to immune checkpoint inhibitor-based immunotherapy. The IPS of patients was obtained from the Cancer Immunome Atlas (https://tcia.at/home) (30).

### Construction and validation of a predictive nomogram

To validate the ability of the ICI score to predict prognosis, a nomogram was constructed based on the clinical features like the Gleason score, age, and T stage of patients obtained from TCGA-PRAD. The calibration method was used to verify the nomogram.

### Statistical analysis

R package V 4.0.5 was used to perform all statistical analyses. The comparisons between the two groups were performed using the Wilcoxon test. The Kruskal–Wallis test was used for comparing more than three groups. The Kaplan–Meier curve was conducted to generate survival differences with the log-rank test in patients with PC. Pearson correlation analysis was used to determine the correlation coefficient. *P*<0.05 was considered statistically significant. Chi-square test was used to analyze the correlation between ICI score subgroups and somatic mutation frequency. Pearson correlation analysis was used to calculate the correlation coefficient. Two-tailed *P*<0.05 was considered statistically significant. False discovery rate was used to correct the *P*-value.

## Results

### The ICI landscape in the TME of PC

CIBERSORT and ESTIMATE algorithms in the R package were used to determine the ICI landscape of patients with PC by calculating the immune score, stromal score, and the content of 22 immune cells (**Supplementary Table S2**) (17, 18). The data of 1,099 patients with PC matched with ICI profile were retrieved from five CPGEA, GEO (GSE116918, GSE70768, and GSE70769), and TCGA. These patients were classified into three distinct ICI subgroups by unsupervised clustering using the “ConsesusClusterPlus” R package (**Supplementary Figures S2A–F**).

**Figure 1:**
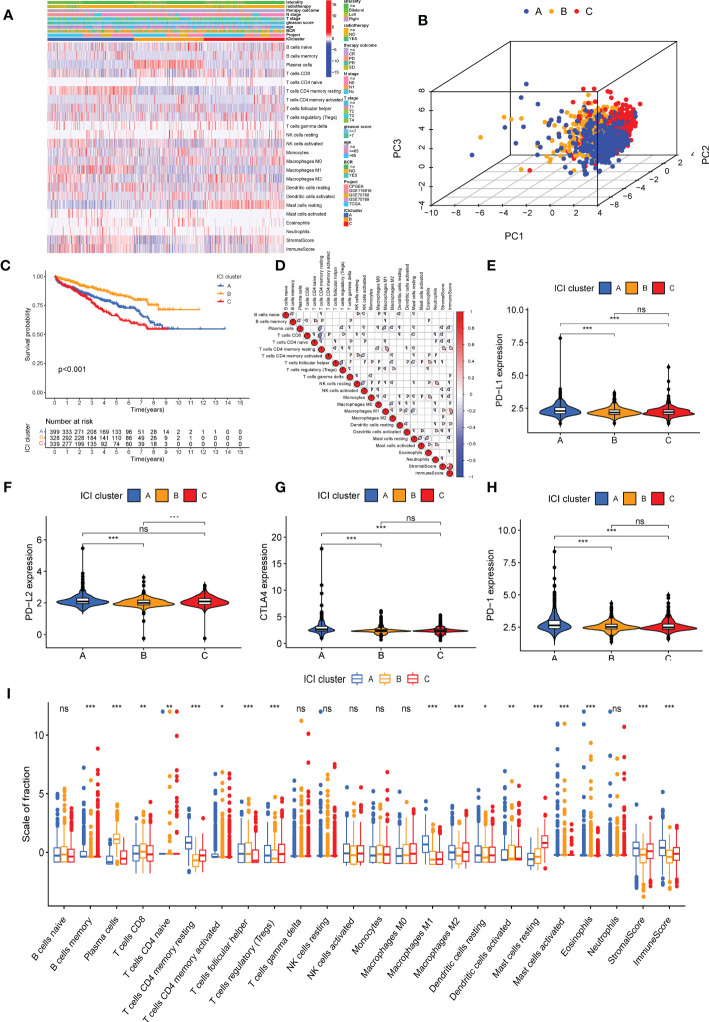
Immune cell infiltration (ICI) cluster subtypes of prostate cancer (PC). **(A)** The heat map shows the distribution of 22 immune cells, immune score, and stromal score in three distinct ICI cluster subtypes. **(B)** Three-dimensional diagram of principal component analysis of ICI clusters. **(C)** Kaplan–Meier curve was used to predict the biochemical recurrence-free survival of patients in four ICI clusters (log-rank test, *P*< 0.001). **(D)** Immune cellular correlation of the ICI score subtypes. Difference in the expression of *PD-L1* **(E)**, *PD-L2* **(F)**, *PD-1* **(G)**, and *CTLA4* **(H)** in patients in three ICI clusters. **(I)** Difference in the infiltration of 22 immune cells, immune score, and stromal score in three distinct ICI clusters. *P< 0.05, **P< 0.01, ***P< 0.001. ns, no significance. CR, complete remission; PR, partial remission; SD, stable disease; PD, progressive disease.

A significant difference in BCR-free survival was observed in three ICI clusters (**Figures 1A, B**). The prognosis of patients in ICI cluster B was better (log-rank test, *P* = 0.002; **Figure 1C**). The immune cell composition of the TME was compared with explore the intrinsic biological differences which led to differences in clinical phenotypes. The landscape of immune cell interaction in the TME and the proportion of ICI were visualized using a correlation coefficient circle plot (**Figure 1D** and **Supplementary Figure S3A**) and the proportion of ICI (**Supplementary Figure S3D**). The expression of four important immune checkpoints [programmed cell death ligand (PD-1), programmed cell death 1 ligand 1 (PD-L1), programmed cell death 1 ligand 2 (PD-L2), and CTLA4)] was analyzed in patients in the three ICI clusters. The results revealed that the expression of four immune checkpoints was highest in patients in ICI cluster A (**Figures 1E–H**). Finally, high infiltration of plasma cells, CD8 T cells, T follicular helper cells, activated dendritic cells, and activated mast cells was observed in patients in ICI cluster B. In the patients in ICI cluster A, increased infiltration of memory-resting CD4 T cells, activated CD4 T cells, M1 macrophages, resting dendritic cells, and eosinophils and high stromal and immune scores were observed. The patients in ICI cluster C were characterized by a high infiltration of T regulatory (Tregs) cells, M2 macrophages, and resting mast cells (**Figure 1I**).

### Identification of gene subtypes based on DEG

**Figure 2:**
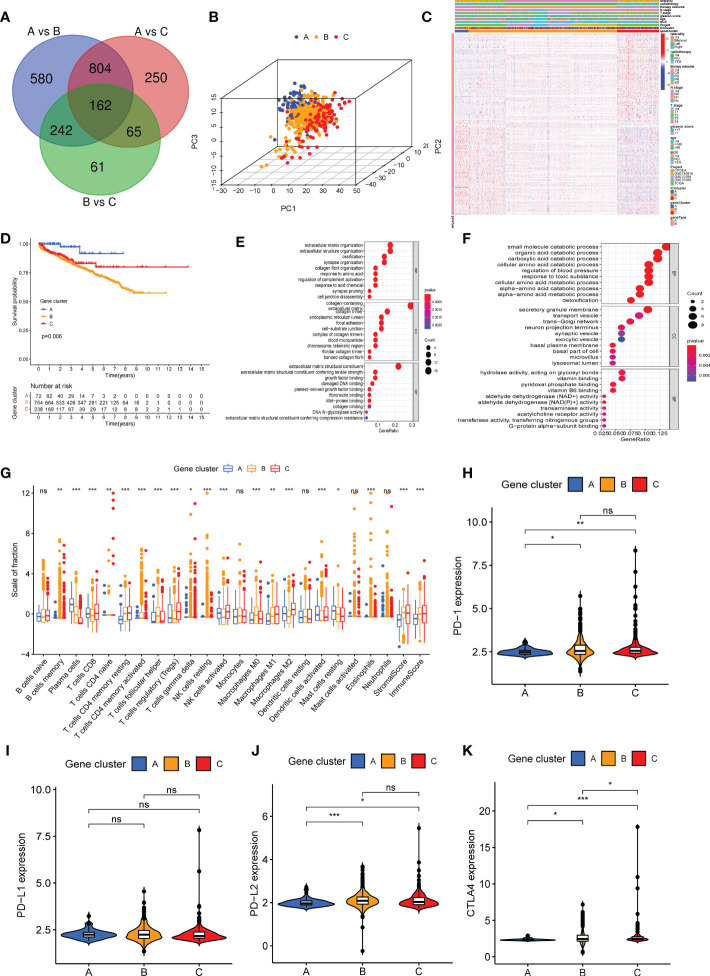
Three different genetypes were stratified based on overlapping differentially expressed genes in three immune cell infiltration (ICI) clusters. **(A)** A total of 162 DEGs were screened from three ICI clusters. **(B)** A three-dimensional diagram of principal component analysis shows the distribution of the three gene clusters. **(C)** The heat map shows the distribution of 22 immune cells, immune score, and stromal score in the three gene clusters. **(D)** Kaplan–Meier curve was used to analyze the biochemical recurrence prognosis among three gene clusters (log-rank test, *P* = 0.006). **(E, F)** Gene Ontology enrichment analysis of genes in ICI genetype A **(E)** and ICI genetype B **(F)**. **(G)** The infiltration of 22 immune cells, immune score, and stromal score in patients in three gene clusters. **(H–K)** Difference in the expression of *PD1* **(H)**, *PD-L1* **(I)**, *PD-L2* **(G)**, and *CTLA4* **(K)** in three gene clusters. *P< 0.05, **P< 0.01, ***P< 0.001, ns, no significance.

The “Limma” R package was used to determine transcriptomic differences in these ICI subtypes for exploring the biological features of distinct immunophenotypes and DEG (22). Based on 162 overlapping DEGs (**Supplementary Table S3** and **Figure 2A**), unsupervised clustering was performed to group 1,099 patients with PC into three genomic clusters such as ICI gene clusters A, B, and C (**Supplementary Figures S4A–F** and **Figure 2B**). Then, we used the correlation analysis method to identify the relationship between DEGs and gene cluster. About 61 gene signatures that were positively correlated with the gene cluster were termed as the ICI genetype A, and the rest of the DEGs were called the ICI genetype B (**Supplementary Table S4**). The “Boruta” R package was used to remove the redundant genes and reduce dimensionality in the ICI genetypes A and B (26). A heat map was constructed using 130 significant DEGs to show the transcriptomic profile (**Figure 2C**) annotated by the “clusterProfiler” R package (31). The Kaplan–Meier curve was used to predict the prognosis of patients in ICI gene clusters. The patients in gene cluster A had a favorable prognosis, whereas the prognosis of patients in gene cluster B was poor (log-rank test, *P* = 0.006, **Figure 2D**). The gene ontology (GO) enrichment analysis of the biological processes enriched by ICI genetypes A and B are summarized in **Figures 2E, F**, respectively. The infiltration of 22 immune cells was analyzed in all samples. The results revealed high infiltration of plasma cells, activated dendritic cells, and resting mast cells in the ICI gene cluster A. High infiltration of memory B cells, activated memory CD4 T cells, T follicular helper cells, gamma delta T cells, resting NK cells, M0 macrophages, eosinophils, and stromal score was observed in patients in ICI gene cluster B. High infiltration of CD8 T cells, naive CD4 T cells, memory-resting CD4 T cells, Tregs cells, activated NK cells, M1 macrophages, and M2 macrophages and high immune score were observed in patients in ICI gene cluster C (**Figure 2G**). Furthermore, the expression of immune checkpoints PD1, PD-L1, PD-L2, and CTLA4 was low in ICI gene cluster A (**Figures 2H–K**).

### Screening hub gene of DGEs

**Figure 3:**
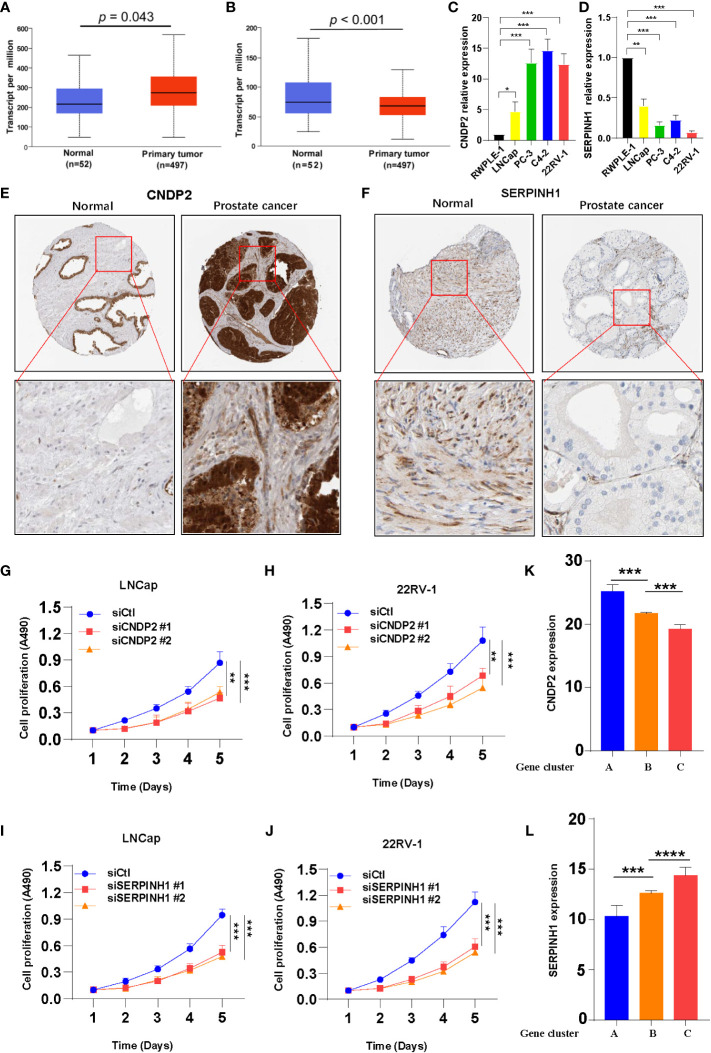
Two hub genes were screened from 162 overlapping differentially expressed genes. **(A)** Increase in *CNDP2* mRNA expression in prostate cancer (PC) tissues compared with normal tissues (*P*< 0.05; T, tumor; N, normal). **(B)** Decrease in *SERPINH1* mRNA expression in PC tissues compared with normal tissue (*P*< 0.05; T, tumor; N, normal). **(C, D)** qPCR results show the expression of *CNDP2* **(C)** and *SERPINH1* **(D)** in immortalized prostate cell lines (RWPE-1) and PC cell lines (LNCap, PC-3, C4-2, and 22RV-1). **(E, F)** Immunohistochemistry results showing the expression of si*CNDP2* **(E)** and *SERPINH1* **(F)** in prostate tumor tissue and normal prostate tissue. **(G, H)** The results of the cell proliferation assay show that si*CNDP2* could suppress the proliferation of LNCap **(G)** and 22RV-1 **(H)**. **(I, J)** The results of the cell proliferation assay show that *siSERPINH1* could suppress the proliferation of LNCap **(I)** and 22RV-1 **(J)**. **(K)** *CNDP2* expression was highest in gene cluster **(A, L)** *SERPINH1* expression was the highest in gene cluster **(C)** *P< 0.05, **P< 0.01, ***P< 0.001, ****P< 0.0001.

Search Tool for the Retrieval of Interacting Genes/Proteins (STRING, https://cn.string-db.org/) database was used to construct the protein–protein interaction network based on 162 DEGs. Two hub genes, *i*.*e*., *CNDP2* and *SERPINH1*, were identified (**Figure S3B**) (32). To study the expression of *CNDP2* and *SERPINH1* in PC, *CNDP2* and *SERPINH1* mRNA expressions in normal prostate and PC tissues were retrieved from the TCGA database. The results revealed that *CNDP2* expression was high in PC compared with normal prostate tissues, and high *SERPINH1* expression was observed in normal prostate tissues compared with PC tissues (**Figures 3A, B**). The qPCR results revealed that the expression of *CNDP2* was high in four PC cell lines (LNCap, PC-3, C4-2, and 22RV-1), and the expression of *SERPINH1* was high in immortalized prostate cell lines like RWPE-1 compared with four prostate cancer cell lines (**Figures 3C, D**). Furthermore, IHC was performed to study the level of *CNDP2* and *SERPINH1* proteins in PC and normal tissue. The results revealed high *CNDP2* expression and low *SERPINH1* expression in PC tissues compared with normal tissues (**Figures 3E, F**).

The expression of *CNDP2* and *SERPINH1* was knocked down using siRNA (si*CNDP2* and si*SERPINH1*) in LNCap and 22RV-1, and the effects of the knockdown are shown in **Figure S3C, D**. Knockdown of *CNDP2* and *SERPINH1* expression inhibited the proliferation of prostate cancer cell lines (**Figures 3G–J**). The expression of two hub genes was evaluated in the three gene clusters, and the results revealed high *CNDP2* expression in patients in gene cluster A and that the expression of *CNDP2* was low in gene cluster C (**Figure 3K**). Furthermore, the expression of *SERPINH1* was higher in gene cluster C compared with gene clusters A and B (**Figure 3L**). The analysis performed using the TIMER database revealed a positive correlation between B cells, CD8+ T cells, dendritic cells, and *SERPINH1* (**Supplementary Figure S5**).

**Figure 4:**
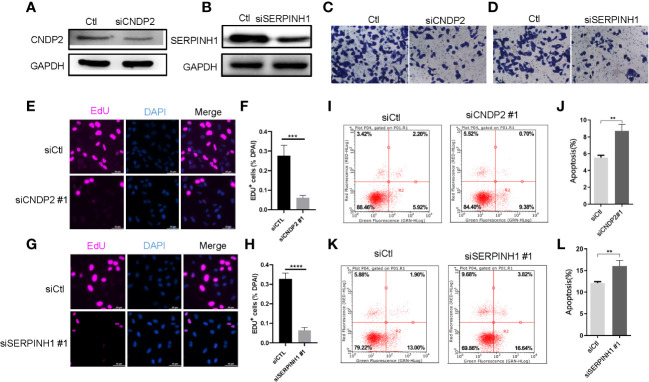
*CNDP2* and *SERPINH1* promotes prostate cancer cell proliferation, migration, and invasion. **(A, B)** Western blot assay showing that si*CNDP2* and si*SERPINH1* were obviously reduced at the protein level. **(C, D)** Transwell invasion assay showing that *CNDP2* and *SERPINH1* increased LNCap cell invasion. **(E–H)** EdU assay showing that *CNDP2* and *SERPINH1* increased LNCap cell proliferation (*P<* 0.001). **(I–L)** Apoptosis assay showing that *CNDP2* and *SERPINH1* reduced LNCap cell apoptosis. ** *P*< 0.01, *** *P*< 0.001, *** P< 0.0001.

According to western blotting (WB) assay, we further observed that *siCNDP2* and *siSERPINH1* were obviously less expressed at the protein level (**Figures 4A, B**). According to the Transwell assay, we found that *siCNDP2* and *siSERPINH1* reduced the invasion function of LNCap (**Figures 4C, D**). Moreover, based on the EdU assay, we found that *CNDP2* and *SERPINH1* contribute to cell proliferation (**Figures 4E–H**). We also utilized flow cytometry to explore the apoptosis of cell, and we observed that the knockdown of CNDP2 and SERPINH1 contributes to more LNCap cell apoptosis (**Figures 4I–L**).

### Generation of ICI score

**Figure 5:**
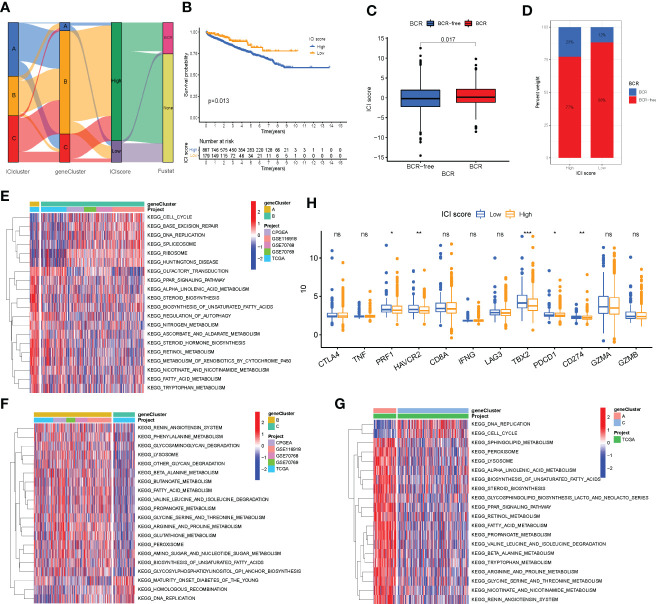
Immune cell infiltration (ICI) score pattern was constructed. **(A)** Sankey diagram shows the correlation between gene cluster, ICI score, and biochemical recurrence (BCR) status of prostate cancer patients. **(B)** The Kaplan–Meier curve was used to predict the prognosis of the ICI score (log-rank test, *P* = 0.013). **(C, D)** Higher ICI score in BCR patients than those who are BCR-free (log-rank test, *P* = 0.017). **(E–G)** Pathways enriched by three ICI clusters using Kyoto Encyclopedia of Genes and Genomes pathway enrichment analysis and identified by the “GSVA” R package. **(H)** Expression of immune-checkpoint genes and immune-activity genes in patients in the high- and low-ICI-score subgroups. *P< 0.05, **P< 0.01, ***P< 0.001. ns, no significance.

**Figure 6:**
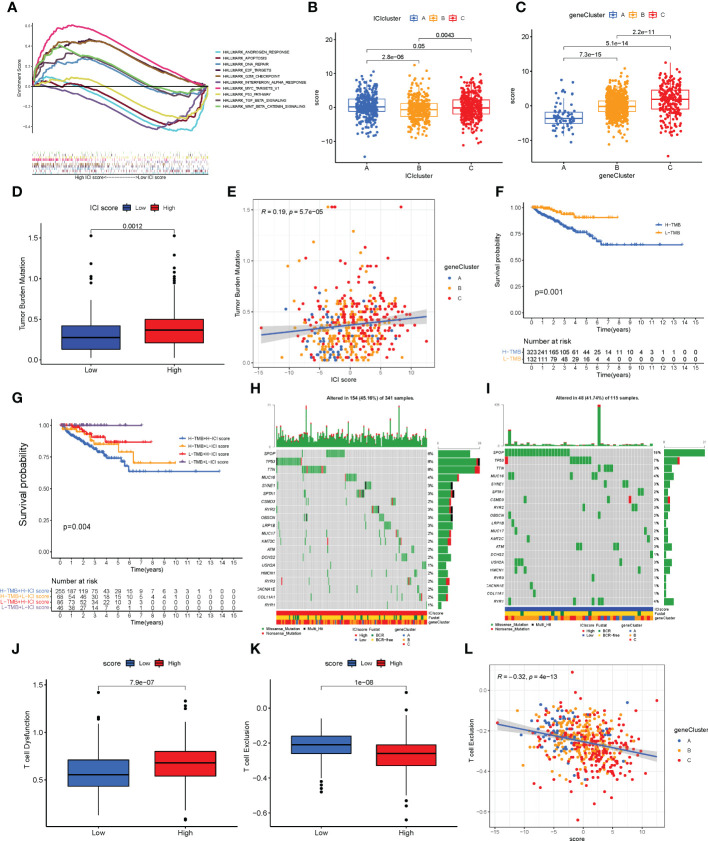
Characteristics of the immune cell infiltration (ICI) score pattern. **(A)** Gene set enrichment analysis in patients in the low- and high-ICI-score subgroups. **(B, C)** ICI score distribution in ICI cluster **(B)** and gene cluster **(C)**. **(D, E)** Patients with high ICI score had a high tumor mutation burden (TMB) value (**D**, *P* = 0.0012), and positive correlation was observed between ICI score and TMB (**E**, *R* = 0.19; *P*< 0.0001). **(F)** Kaplan–Meier curve for low and high TMB subgroups in the ICI score cohort (log-rank test, *P* = 0.001). **(G)** Kaplan–Meier curve for different subgroups (log-rank test, *P* = 0.004). **(H, I)** Top 20 driver genes in patients in the low- **(H)** and high-ICI-score subgroups **(I)**. **(J)** Status of T cell dysfunction in patients in the high- and low-ICI-score subgroups (log-rank test, *P<* 0.0001). **(K, L)** Patients with high ICI score had low T cell exclusion (**K**; log-rank test, *P<* 0.0001), and negative correlation between ICI score and T cell exclusion (**L**; *R* = 0.21; *P*< 0.0001).

PCA was used to calculate the score of gene signatures A and B, and the ICI score was obtained after subtracting the B score from the A score. The ICI scores are listed in **Supplementary Table S5**. A total of 1,099 patients with PC were divided into high- and low-ICI-score groups based on the optimal cutoff value (cutoff value = -2.955722). **Figure 5A** shows the distribution of patients with PC in ICI gene clusters and two ICI score subgroups. Kyoto Encyclopedia of Genes and Genome (KEGG) pathway enrichment analysis was performed to identify the pathways enriched by the three ICI clusters using Gene Set Variation Analysis (GSVA) in the R package (**Figures 5E–G**). Next, we evaluated the prognostic value of the ICI scores using the Kaplan–Meier curve. The results revealed that the patients in the high-ICI-score subgroup had a favorable BCR-free survival compared with the patients in the low-ICI-score subgroup (log-rank test, *P* = 0.013, **Figure 5B**). Furthermore, the “limma” R package was used to study the correlation between ICI score and BCR, and the results suggest that patients with high ICI scores had a higher chance of BCR occurrence (**Figures 5C, D**). Based on previous studies, the genes CD274, *CTLA4*, *HAVCR2*, *LAG3*, and *PDCD1* were selected as immune-checkpoint-relevant gene signatures and the genes *CD8A*, *GZMA*, *GZMB*, *IFNG*, *PRF1*, *TBX2*, and *TNF* as immune-activity-related gene signatures to analyze the tolerance and immune activity of patients in all groups (33, 34). Wilcoxon test was performed, and the results revealed a significant increase in the expression of *HAVCR2*, *PDCD1*, *CD274*, *PRF1*, and *TBX2* in patients in the low-ICI-score group compared with patients in the high-ICI-score group (**Figure 5H**). Gene set enrichment analysis (GSEA) showed significant enrichment of the WNT-β and TGF-β signaling pathways in the high-ICI-score subgroup, whereas the androgen receptor and interferon alpha response pathways were enriched in patients in the low-ICI-score subgroup (**Figure 6A**). The results of the Kruskal-Wallis test revealed a high correlation between ICI cluster A, gene cluster C, and ICI score (**Figures 6B, C**). We further used ROC curve to assess ICI cluster, gene cluster, and ICI score (**Supplementary Figure S7G**).

### The relationship between somatic mutation and the ICI scores in PC

TMB is an important biomarker of cancers. Studies have shown an association between high TMB and high CD8+ T cell infiltration in PCs (5, 35). The TMB values are listed in **Supplementary Table S6**. Considering the significant value of TMB in clinical settings, the correlation between the ICI scores and TMB was evaluated to understand the genetic imprints of each ICI score subgroup. First, the ICI high and low scores and TMB values of patients were compared. As shown in **Figure 6D**, high TBM was observed in patients in the high-ICI-score subgroup compared with patients in the low-ICI-score subgroup (Wilcoxon test, *P* = 0.0012). Furthermore, a positive correlation was observed between TMB and ICI score (Spearman coefficient: *R* = 0.19, *P*< 0.001, **Figure 6E**). Next, the patients with PC were divided into subgroups based on the TMB immune set point (30). As shown in **Figure 6F**, the prognosis of patients with high TMB value was poor compared with patients with low TMB value (log-rank test, *P* = 0.001). Based on the contraindicatory prognostic value of ICI score and TMB, the synergy between TMB and ICI score to divide the patients based on the prognosis was assessed. The patients were divided into four subgroups: high TMB + high ICI score, high TMB + low ICI score, low TMB + high ICI score, and low TMB + low ICI score subgroups. The stratified BCR-free survival analysis showed that TMB status does not affect ICI score predictions. As shown in **Figure 6G**, the ICI score demonstrated a significant difference in BCR-free survival in patients with high and low TMB scores. The patients in low TMB + low-ICI-score subgroups had better prognosis (log-rank test, *P* = 0.004).

Moreover, the distribution of somatic variants of driver genes was evaluated between the low- and high-ICI-score subgroups using the “maftools” R package. The mutation frequency of the top 20 driven genes is shown in **Supplementary Figure S6A**. The mutation frequency was high in *SPOP*, *TTN*, and *TP53* in patients with PC, which may indicate some novelty associated with ICI score and immune checkpoint inhibitor therapy (**Supplementary Figure S6B**). The analysis of the co-occurrence of the top 25 mutated genes is shown in **Supplementary Figure S6C**. The top 20 driver genes with a high frequency of mutations in patients in the high- and low-ICI-score subgroups are shown in **Figures 6H, I**.

### The correlation between ICI scores and TIDE

TIDE can provide data-driven gene signatures of T cell dysfunction and exclusion (29). The data on T cell dysfunction and T cell exclusion by TIDE was obtained from the transcriptome data of patients retrieved from TCGA-PRAD. High T cell dysfunction and low T cell exclusion were observed in patients with high ICI scores (**Figures 6J, K**). A negative correlation was observed between the ICI score and T cell exclusion (**Figure 6L**).

### Correlation between ICI score and clinical features and the application of predicting immunotherapy

**Figure 7:**
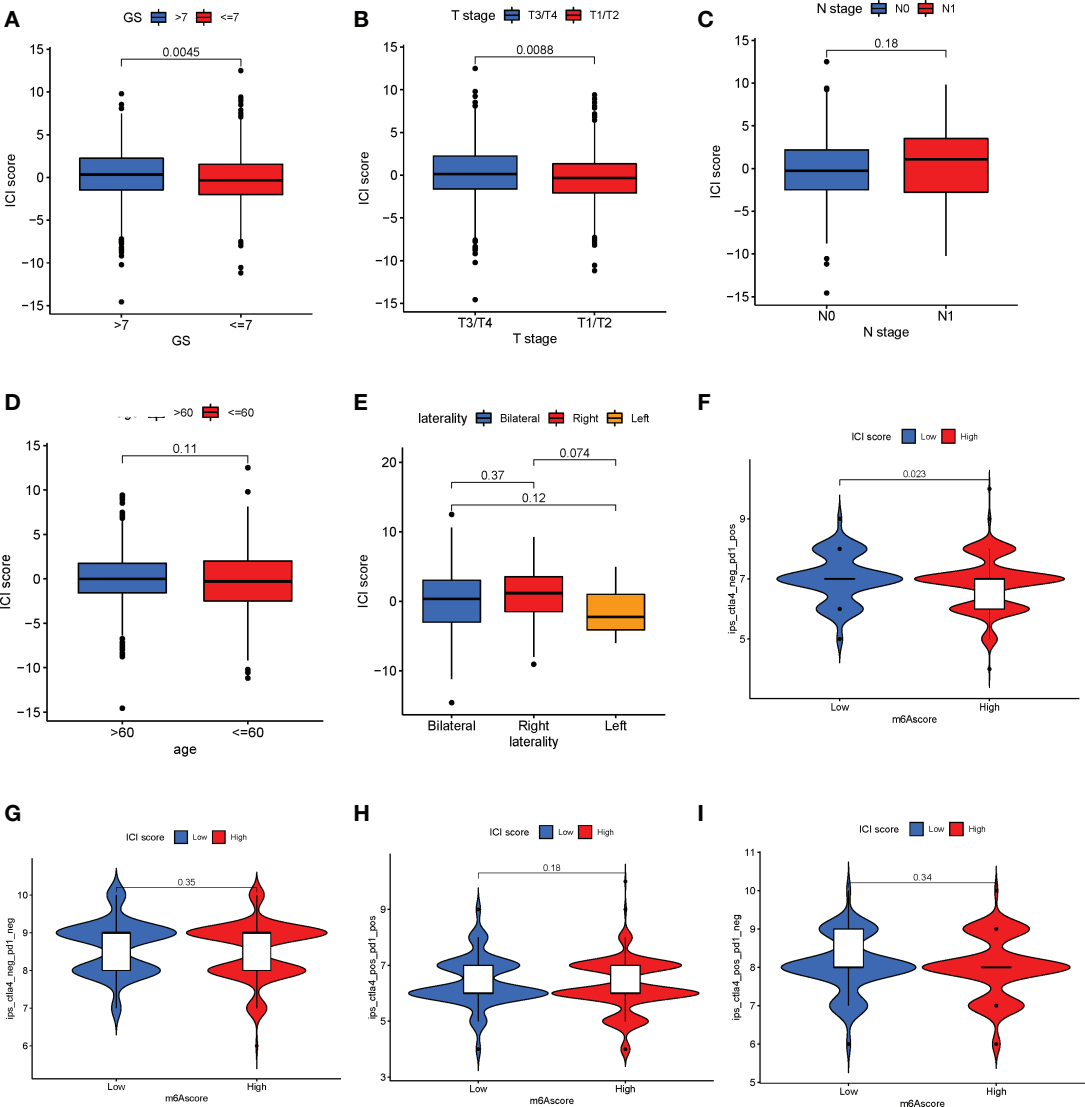
Correlation between immune cell infiltration (ICI) scores and major clinical features of patients with prostate cancer. **(A)** Correlation between the ICI and Gleason scores (Wilcoxon test, *P* = 0.0045). **(B)** Association between T stage and ICI scores (Wilcoxon test, *P* = 0.0088). **(C–E)** The N stage **(C)**, age **(D)**, and laterality € parameters have no significant correlation with the ICI score. **(F)** Patients with a low ICI score have a better immune response to the IPS-CTLA4-neg-PD1-pos immunotherapy (**F**; log-rank test, *P* = 0.023). **(G–I)** No significant difference in immunophenotype scores in patients with high and low ICI scores.

The correlation between ICI score and clinical features such as Gleason score, age, node metastasis (N), and tumor stage (T) in patients with PC was explored. The results revealed that patients with Gleason score >7 (**Figure 7A**) and T3/T4 stage (**Figure 7B**) had a higher ICI score (Wilcoxon test, *P*< 0.01). However, no significant correlation was observed between the N stage (**Figure 7C**), age (**Figure 7D**), laterality **(** **Figure 7E**, Wilcoxon test, *P* > 0.05), and ICI score. These results together indicate a correlation between the ICI score and the progression of PC. As mentioned previously, the patients were divided into high- and low-ICI-score subgroups. Furthermore, the correlation between immune checkpoints and ICI score was determined (30). Based on the ICI score, the patients were divided into four subgroups, including IPS-CTLA4-neg-PD1-neg, IPS-CTLA4-neg-PD1-pos, IPS-CTLA4-pos-PD1-pos, and IPS-CTLA4-pos-PD1-neg. As shown in **Figure 7F**, the patients with the low ICI score had better objective responses to IPS-CTLA4-neg-PD1-pos immunotherapy compared with patients with the high ICI score (Wilcoxon test *P* = 0.023). However, no association was observed between ICI score and the patients in IPS-CTLA4-neg-PD1-neg, IPS-CTLA4-pos-PD1-pos, and IPS-CTLA4-pos-PD1-neg immunotherapy (Wilcoxon test *P* > 0.05, **Figures 7G–I**). For verifying the ICI score of immunotherapy prediction, we collected data of two cohorts, IMvigor210 and GSE78220. We found low ICI score performed well response to immunotherapy (**Supplementary Figures S7H–I**).

**Figure 8:**
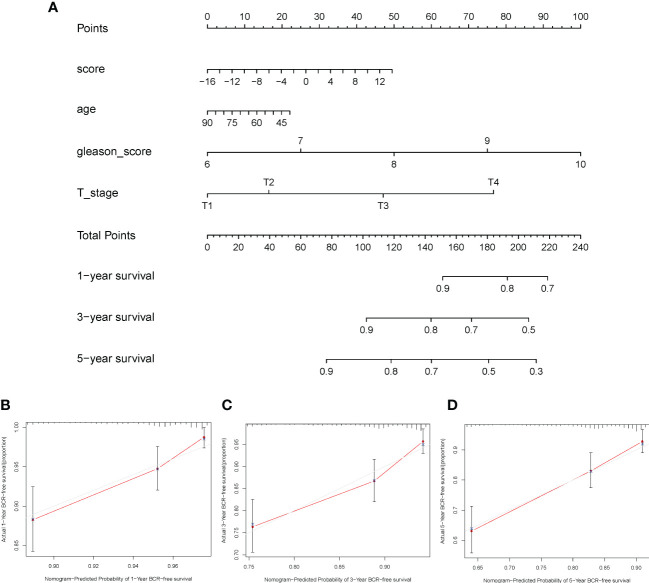
Construction of nomogram for prostate cancer. **(A)** The nomogram was based on immune cell infiltration score, age, Gleason score, and T stage to predict the prognosis of 1, 3, and 5 years. **(B–D)** The 1-year **(B)**, 3-year **(C)**, and 5-year **(D)** calibration curves of the nomogram.

A nomogram based on clinical features was constructed using the “rms” R package to determine the prognostic value of the ICI score. Each parameter (ICI score, age, Gleason score, and T stage) was assigned a point, and the total points were computed. Based on the total score, 1-, 3-, and 5-year BCR-free survival rates were predicted (**Figure 8A**). The calibration plot validated that the nomograms could predict the prognosis of patients based on ICI scores (**Figures 8B–D**).

### Tumor purity and stromal score among ICI clusters, gene clusters and ICI score

According to ESTIMATE algorithms, ICI cluster B (Wilcoxon test *P*< 0.001), gene cluster A (Wilcoxon test, *P*< 0.001) and high-ICI-score patients performed higher tumor purity (Kruskal–Wallis test *P*< 0.001). In addition, ICI cluster B (Wilcoxon test *P*< 0.001), gene cluster A (Wilcoxon test, *P*< 0.001), and high-ICI-score patients exhibited a lower stromal score (Kruskal–Wallis test, *P*< 0.001) (**Supplementary Figures S7A–F**).

## Discussion

PC is a highly heterogeneous and complex cancer. In total, 60%–90% of the patients with PC have multiple cancer foci (36). The traditional treatment strategies for PC involve ADT and chemotherapy. However, drug resistance commonly occurs with anticancer treatment strategies available. Several advancements have been made in immunotherapy in the past few decades. Immunotherapy has been used in the treatment of multiple cancers like melanoma, kidney cancer, non-small cell lung cancer, and PC (37). However, TME can alter the characteristics of tumors and aid immunotherapy (38). Sipuleucel-T was the first novel immunotherapeutic drug approved by the FDA for the treatment of patients with PC (7). However, compared with immunologically responsive cancers, the prognosis of patients with PC receiving immunotherapy are not encouraging (12). Furthermore, PC has traditionally been deemed an immunologically “cold” tumor with complicated TME, thus altering the sensitivity of PC to immunotherapy. Therefore, it is necessary to accurately identify ICI subtypes which could aid in designing personalized therapy. In this study, we developed a method to quantify the comprehensive ICI landscape for patients with PC. Our results will provide a prognostic model which uses the ICI score to predict the immunotherapy response and prognosis of patients.

The TME consists of tumor cells, infiltrated immune cells, stromal cells, extracellular matrix molecules, and pro-inflammatory factors (39). Furthermore, TME can alter the patient’s response to immunotherapy (38). A previous study has demonstrated that tumor cells can activate B cells and promote the secretion of immunoglobulin to inhibit tumor growth (39). CD8+ T cells are associated with immune responses and are a major immune cell type that aids in suppressing tumor growth (40). In this study, we have used publicly available databases to retrieve data on 1,099 patients with PC. The patients could be divided into three distinct ICI subgroups. Our results show increased infiltrations of plasma cells, CD8 T cells, T follicular helper cells, activated dendritic cells, and activated mast cells. Furthermore, there was a decrease in the infiltration of M2 macrophages, Tregs, and resting mast cells, which were significantly correlated with a good prognosis. Our results are consistent with the results of previous studies (41, 42). The status of immune cells affects the anti-tumor response and can aid in improving the response of patients to immunotherapy. Previous studies have shown some benefits of immunotherapy in the treatment of patients with PC (43, 44). However, compared with immune-responsive cancers, the outcomes of immunotherapy in patients with PC are unsatisfactory (12). This suggests that immunophenotypes of tumors cannot categorically predict the efficacy of immunotherapy. Various factors, like the components of TME, chemokines, and cytokines, can alter the efficacy of immunotherapy in the treatment of patients with PC. These molecular changes in tumor cells could impact interactions between infiltrated immune cells, which alters the stability between immune activity and immune tolerance (45).

In our study, we proposed that immune-related gene expression patterns and characterization of ICI profiles could be used to develop personalized therapeutic strategies for patients with PC. Our study focuses on understanding the molecular mechanism regulating the immune cells; thereby, we obtained the immune-related DEGs among three ICI clusters. The patients were divided into three gene clusters based on DEGs. The patients in gene cluster C had a high stromal and immune score and increased infiltration of CD8 T cells, naive CD4 T cells, memory-resting CD4 T cells, along with high PD-1 and CTLA4 expression, thus suggesting that these patients had an immune-hot phenotype (46, 47). We hypothesize that patients in gene cluster C may benefit from immunotherapy. Furthermore, the patients in gene cluster B had a lower immune score compared with patients in gene cluster C and low infiltration of the immune cells in the tumor, which indicate that patients had an immune-cold phenotype. Previous studies have shown that TME alters the prognosis of patients with cancer (48, 49). Consistent with previous studies, our results show that the prognosis of patients in gene cluster C was good. The activated lymphocytes in patients in gene cluster C could trigger infiltration of more immune cells in the tumor, thereby improving response to immunotherapy. Since our results are similar to previous studies, these gene clusters could aid in designing immunotherapeutic strategies. Furthermore, we identified two hub genes from 162 DEGs, which helped classify the patients in the immune cluster by influencing the infiltration of immune cells in tumors. The analysis conducted using the TIMER database revealed a positive correlation between the hub gene *SERPINH1* and immune cells like B cells, CD8+ T cells, and dendritic cells. Furthermore, the expression of *SERPINH1* was high in patients in gene cluster C. Previous studies have shown that *CNDP2* is pro-oncogene, involved in cell proliferation and metastasis in ovarian cancer *via* the PI3K/AKT pathway (50). Furthermore, *SERPINH1* expression was associated with poor prognosis in patients with breast cancer, stomach adenocarcinoma, and esophageal carcinoma (51). According to our *in vitro* experiments, we found that *CNDP2* and *SERPINH1* promote tumor invasion and cell proliferation and reduce cell apoptosis in prostate cancer. Moreover, our results also revealed that the expression of hub genes *CNDP2* and *SERPINH1* significantly increases the proliferation of PC cells. These results together indicate that *CNDP2* and *SERPINH1* play a cancer-promoting effect and may affect the infiltration of immune cells in PC, which could be the underlying factor associated with better prognosis of patients in gene cluster C compared with patients in gene cluster B. Furthermore, *in vitro* or *in vivo* experiment would further validate the immunogenic role of CNDP2 and SERPINH1 in the next step.

TME is heterogenous; hence, it is necessary to study the ICI patterns for all cancers. Based on tumor subtype-specific biomarkers, an effective individual-based model of pattern has been constructed in breast cancer and head and neck cancer (52, 53). We used the Boruta algorithm in the R package to identify ICI patterns based on ICI scores generated by potential “subtype biomarkers.” Based on the Boruta screen, feature genes were further identified from genetypes A and B. PCA was used to extract the PCA value as the genetype score. Therefore, ICI score model may better reflect immune characteristics in PC. GSEA revealed that the WNT-β and TGF-β pathways were enriched in patients in the high-ICI-score subgroups, which may contribute to immune tolerance in PC. Our results show a high expression of immune-checkpoint-related gene signatures and immune-activity-related gene signatures in patients in the low-ICI-score subgroup, indicating that the patients in this group were sensitive to immunotherapy. Several preclinical studies have shown a close correlation between gene mutations and immunotherapeutic response or tolerance (54–56). A comprehensive analysis of ICI scores at the genome level showed a significant difference in the mutation frequency of multiple genes in patients in the high- and low-ICI-score subgroups. Furthermore, our results show a positive correlation between TMB and ICI score (*R* = 0.19). The survival analysis revealed that the prognostic value of the ICI scores was independent of the TMB value. Therefore, ICI score and TMB may indicate a difference in the immune status of PC; thus, ICI score could be a prognosis biomarker for an immunotherapeutic response independent from TMB. Previous studies have shown that patients with high tumor mutation produce more novel tumor antigen, which makes the tumors more susceptible to attack by immune cells (5, 57). Our results show a positive correlation between T cell exclusion and high ICI scores. We hypothesize that a correlation between a high ICI score and high TMB could increase the infiltration of more T cells in PCs. However, high T cell dysfunction was observed in patients in the high-ICI-score subgroup. Despite the high infiltration of T cells in patients with high ICI scores, there was high T cell dysfunction in these patients, which may contribute to poor prognosis in patients with high ICI scores.

Furthermore, a correlation between ICI score and clinical features in patients with PC was observed. The results show that patients with Gleason scores >7 and T3/T4 stage had high ICI scores, indicating that high ICI scores may be correlated with the development of PC and poor prognosis. The data on patients treated with immune checkpoint inhibitors were obtained from TCGA-PRAD, and the ICI score was calculated. The analysis revealed that a significant decrease in ICI score was observed in patients with better response to immunotherapy, thereby validating the prognostic value of ICI score. Therefore, patients with low ICI scores could benefit from single-agent immunotherapy. Furthermore, the TGF-β signaling pathway was activated in patients in the high-ICI-score subgroups; therefore, the combined use of immune checkpoint inhibitors and TGF-β blockers could benefit patients with high ICI scores (58, 59). Previous studies have shown that the combined use of immune checkpoint inhibitors and TGF-β blockade was more effective than a single agent. However, additional studies with a larger sample size of patients treated with immunotherapy are required to validate our results. The nomogram has been widely used as a predictive model for multiple cancers (60–62). In this study, a nomogram based on ICI score was constructed, which could effectively predict the prognosis of patients in a clinical setting.

In conclusion, we provide a comprehensive ICI landscape of patients with PC and insights into the immune response of patients with PC. The distinct subtypes of the ICI model impact the complex tumor therapy and heterogeneity. However, more *in vitro* or *in vivo* experiments are needed to further validate our model and the immunogenic role of CNDP2 and SERPINH1. In summary, we comprehensively analyzed the characteristics of ICI gene signature, which could be used in clinical settings for classifying patients and designing personalized immunotherapy strategies for patients.

## Data availability statement

The original contributions presented in the study are included in the article/**Supplementary Material**. Further inquiries can be directed to the corresponding authors.

## Author contributions

All authors contributed to the conceptualization and design of the study. WW, XW, CL, and HL made substantial contributions to the acquisition of data, analysis, and interpretation of data and drafted the manuscript. LW, QW, WA, and CX revised the manuscript critically for important intellectual content. WL, YZ, and XC responded for statistic and quality review. All authors read and approved the final manuscript.

## Supplementary material

Supplementary Figure 1 Flowchart of the analysis based on bulk of prostate cancer (PC) cohorts. Five independent PC cohorts were combined into a large PC cohort by R “ComBat”. The expression profile of 1,099 PC was quantified as 22 immune terms by “CIBERSORT” and divided into three immune cell infiltration (ICI) clusters by principal component analysis (PCA). Three gene clusters were further obtained by PCA based on the expression of differentially expressed genes. The ICI score was established, and we found that high-ICI-score patients show poor clinical features and that biological pathway was associated with poor prognosis of PC poor clinical outcomes.

Supplementary Figure 2 Assessing consensus and defining the number of immune cell infiltration cluster subgroups (optimal *k*) in prostate cancer. **(A–D)** Heat map of the consensus matrix for **(A)** *k* = 2, **(B)** *k* = 3, **(C)** *k* = 4, and **(D)** *k* = 5. **(E)** Cumulative distribution function (CDF) of unsupervised clustering for *k* = 2 to 9. **(E)** Relative change in area under CDF curve for *k* = 2 to 9.

Supplementary Figure 3
**(A)** Interaction of 22 immune cells in prostate cancer (PC) patients. **(B)** Two hub genes were screened from overlapping differentially expressed genes. **(C, D)** Knockdown efficiency of si*CNDP2* **(C)** and si*SERPINH1* **(D)** in the LNCap cell lines. **(E)** Heat map showing the content of 22 immune cells in each sample. **(F)** Infiltration of 22 immune cells between normal and PC.

Supplementary Figure 4 Measuring consensus and determining the number of gene clusters (optimal *k*) in patients with prostate cancer. **(A–D)** Heat map of the consensus matrix for **(A)** *k* = 2, **(B)** *k* = 3, **(C)** *k* = 4, and **(D)** *k* = 5. **(E)** Cumulative distribution function (CDF) of unsupervised clustering for *k* = 2 to 9. (**E**) Relative change in area under the CDF curve for *k* = 2 to 9.

Supplementary Figure 5 Correlation between hub genes and immune cell infiltration in tumor. **(A)** Correlation between *CNDP2* and major immune cells in patients with prostate cancer (PC). **(B)** Correlation between *SERPINH1* and immune cells in patients with PC.

Supplementary Figure 6 Status of somatic mutation in patients with prostate cancer (PC). **(A)** The “maftools” R package was used to analyze the somatic gene mutation in patients with PC. **(B)** A detailed analysis of the variants. **(D)** The interaction between mutated genes.

Supplementary Figure 7 Tumor purity and stromal score in prostate cancer (PC) patients. **(A, B)** Tumor purity **(A)** and stromal score **(B)** of immune cell infiltration (ICI) clusters. **(C, D)** The tumor purity **(C)** and stromal score **(D)** of gene clusters. **(E, F)** Tumor purity **(E)** and stromal score **(F)** of ICI score. **(G)** Receiver operating characteristic curve of ICI cluster, gene cluster, and ICI score. **(H, I)** Immune response of ICI score in IMvigor210 and GSE78220 cohorts. CR, complete remission; PR, partial remission; SD, stable disease; PD, progressive disease.
