# Supplementary figures and images for: Immune microenvironment infiltration landscape and immune-related subtypes in prostate cancer
Source: Front Immunol. 2023 Jan 9;13:1001297. doi: 10.3389/fimmu.2022.1001297 (PMC9868452; doi:10.3389/fimmu.2022.1001297)

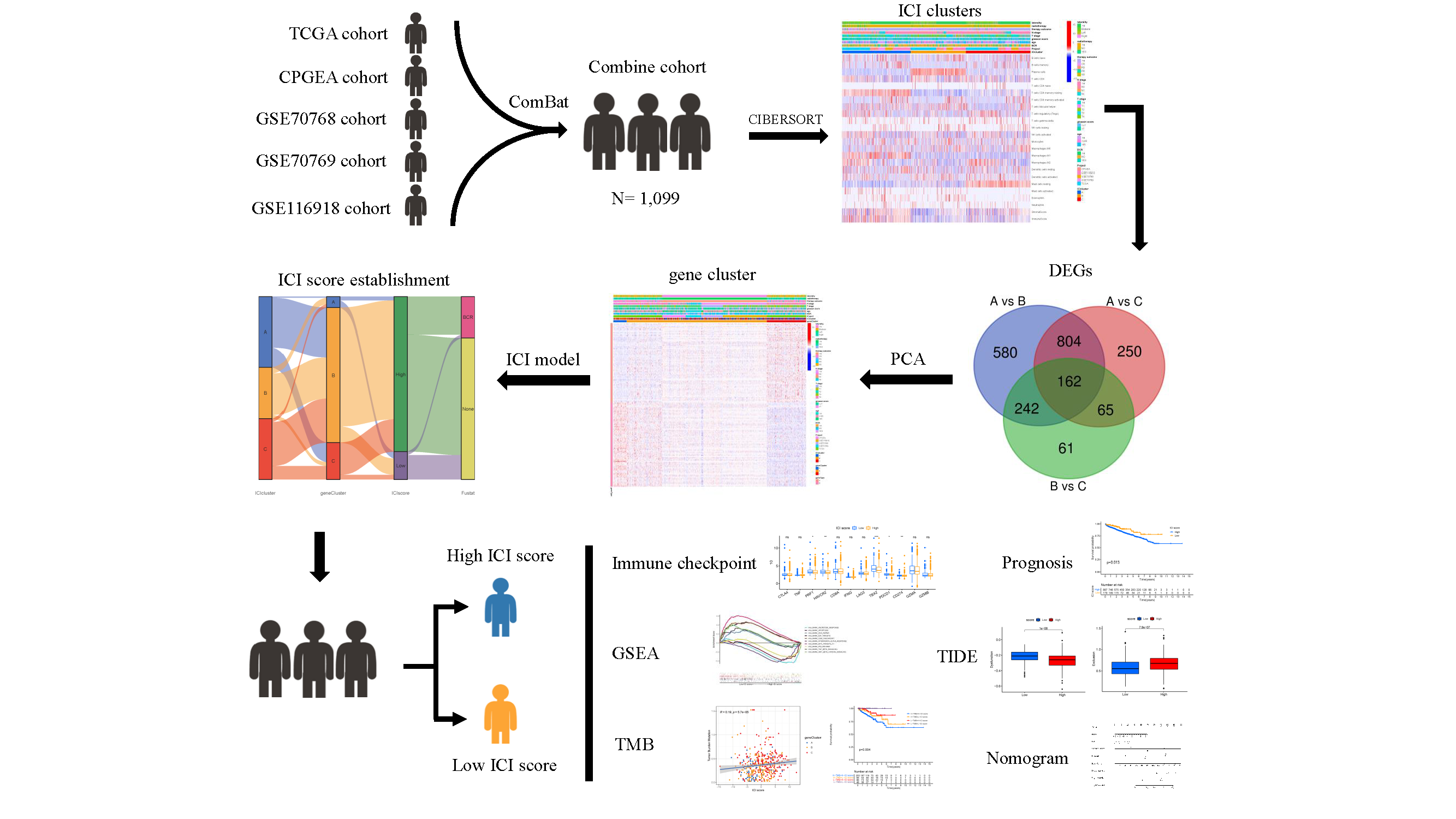

Supplement: Supplementary Figure 1 — Flowchart of the analysis based on bulk of prostate cancer (PC) cohorts. Five independent PC cohorts were combined into a large PC cohort by R “ComBat”. The expression profile of 1,099 PC was quantified as 22 immune terms by “CIBERSORT” and divided into three immune cell infiltration (ICI) clusters by principal component analysis (PCA). Three gene clusters were further obtained by PCA based on the expression of differentially expressed genes. The ICI score was established, and we found that high-ICI-score patients show poor clinical features and that biological pathway was associated with poor prognosis of PC poor clinical outcomes. [file Image_1.tif]

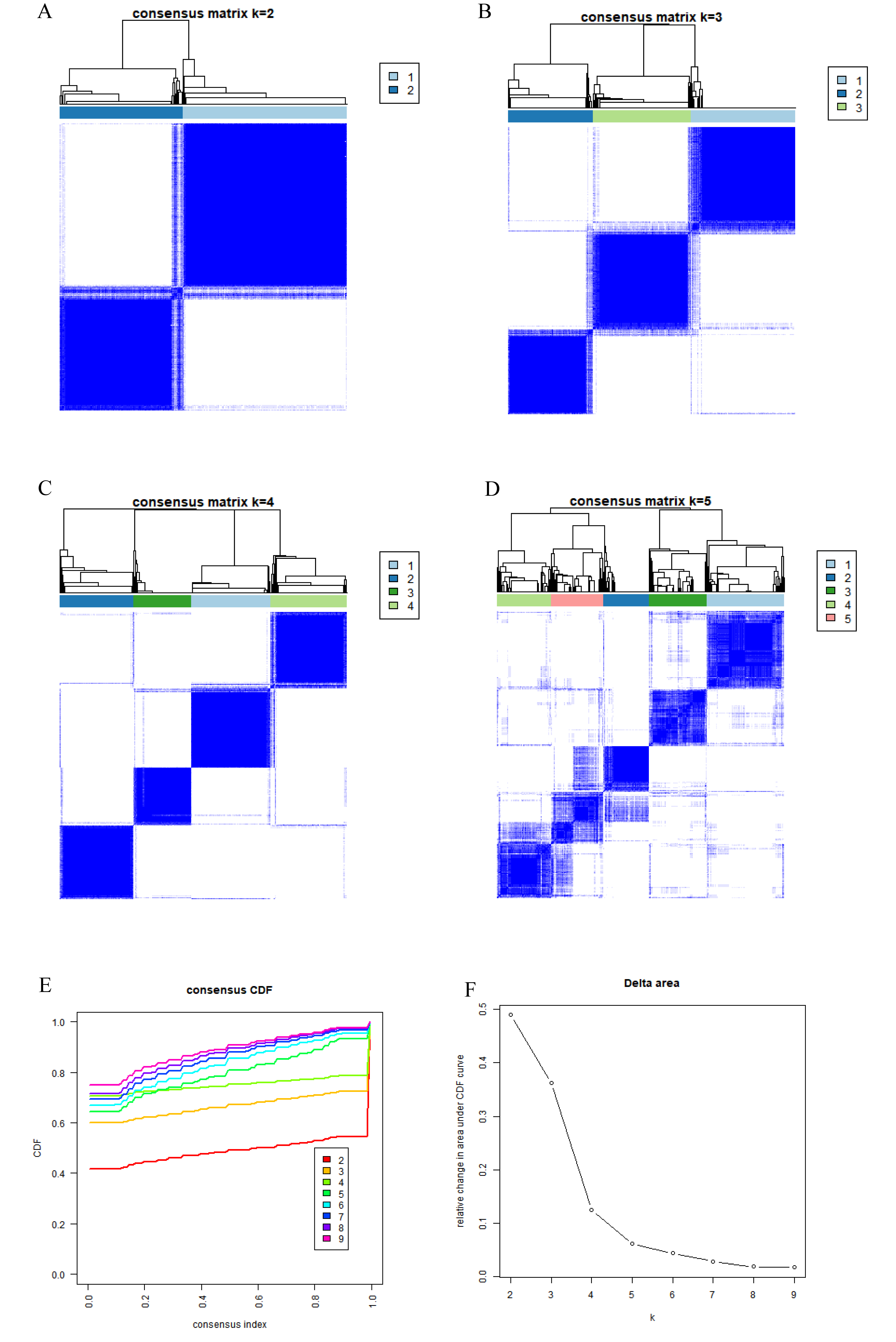

Supplement: Supplementary Figure 2 — Assessing consensus and defining the number of immune cell infiltration cluster subgroups (optimal k) in prostate cancer. (A–D) Heat map of the consensus matrix for (A) k = 2, (B) k = 3, (C) k = 4, and (D) k = 5. (E) Cumulative distribution function (CDF) of unsupervised clustering for k = 2 to 9. (E) Relative change in area under CDF curve for k = 2 to 9. [file Image_2.tif]

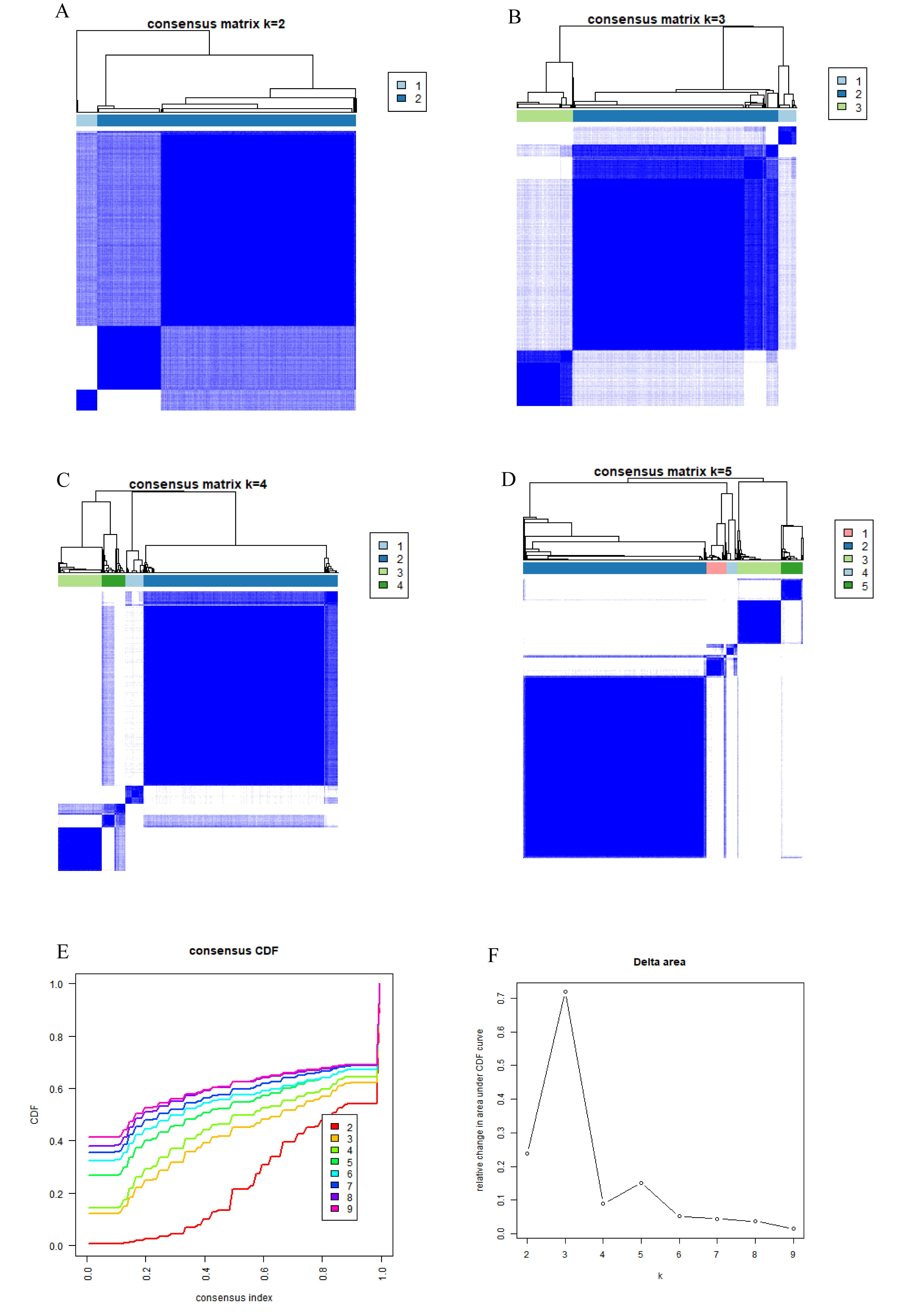

Supplement: Supplementary Figure 4 — Measuring consensus and determining the number of gene clusters (optimal k) in patients with prostate cancer. (A–D) Heat map of the consensus matrix for (A) k = 2, (B) k = 3, (C) k = 4, and (D) k = 5. (E) Cumulative distribution function (CDF) of unsupervised clustering for k = 2 to 9. (E) Relative change in area under the CDF curve for k = 2 to 9. [file Image_4.tif]

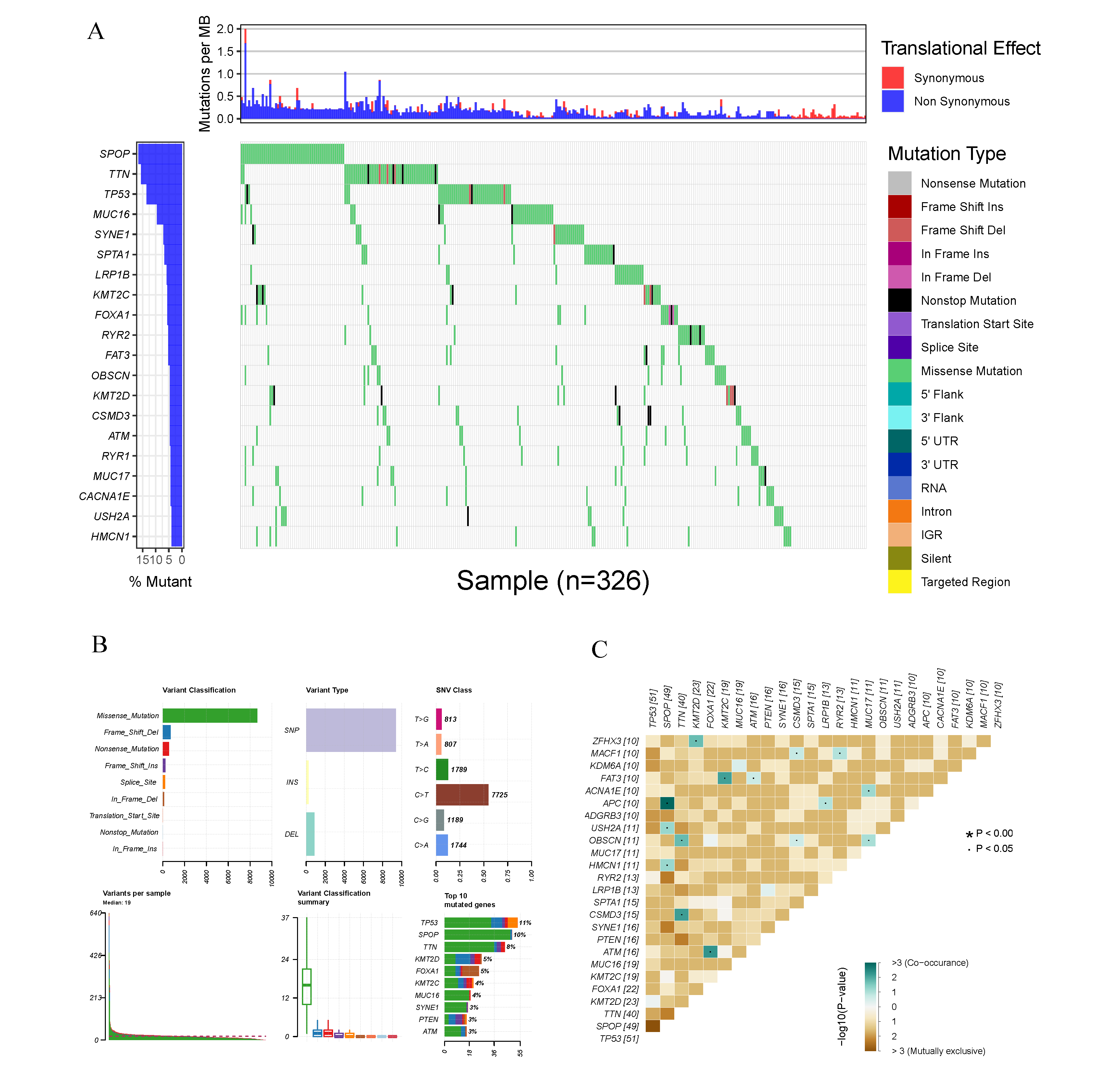

Supplement: Supplementary Figure 6 — Status of somatic mutation in patients with prostate cancer (PC). (A) The “maftools” R package was used to analyze the somatic gene mutation in patients with PC. (B) A detailed analysis of the variants. (D) The interaction between mutated genes. [file Image_6.tif]

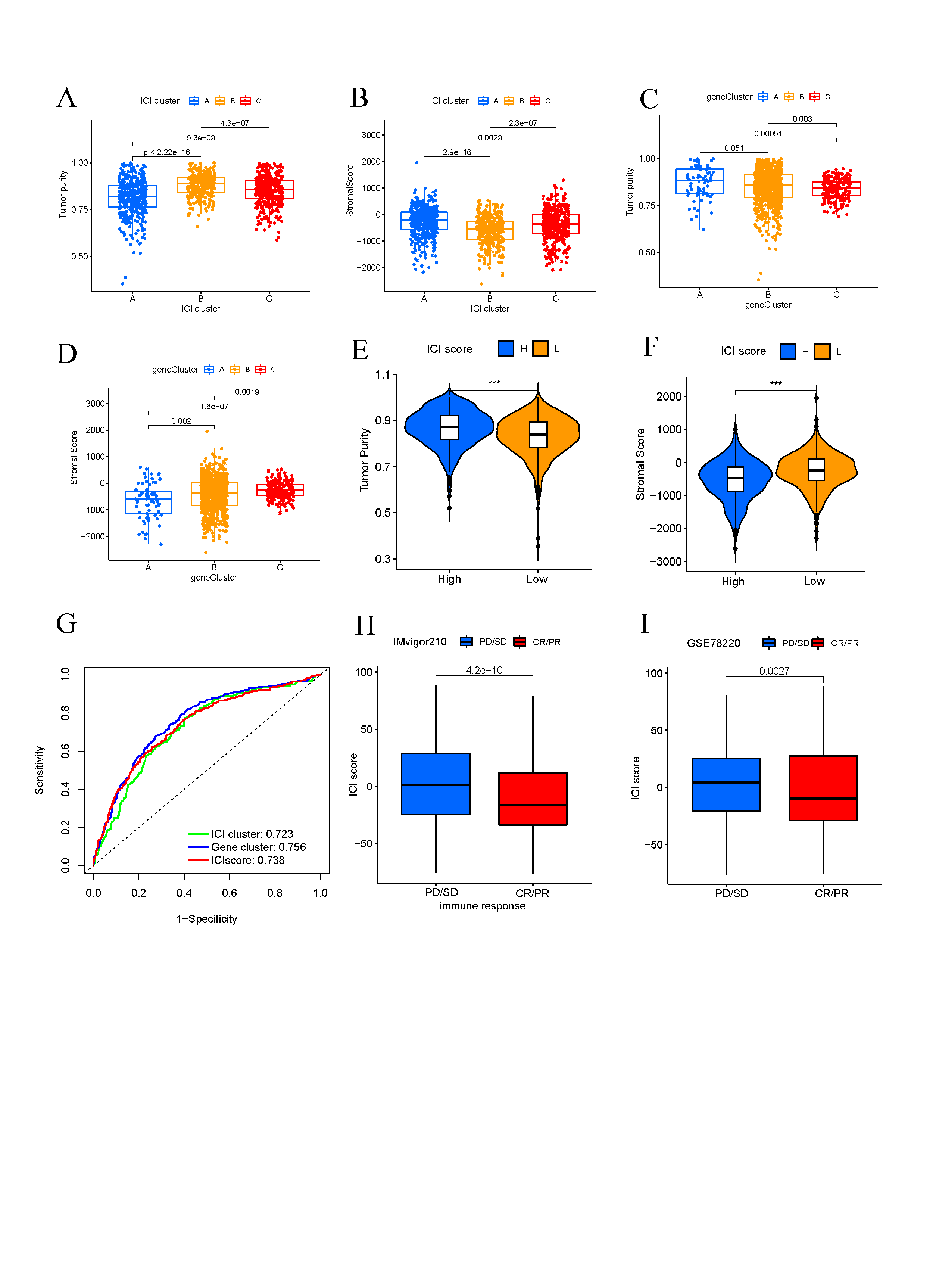

Supplement: Supplementary Figure 7 — Tumor purity and stromal score in prostate cancer (PC) patients. (A, B) Tumor purity (A) and stromal score (B) of immune cell infiltration (ICI) clusters. (C, D) The tumor purity (C) and stromal score (D) of gene clusters. (E, F) Tumor purity (E) and stromal score (F) of ICI score. (G) Receiver operating characteristic curve of ICI cluster, gene cluster, and ICI score. (H, I) Immune response of ICI score in IMvigor210 and GSE78220 cohorts. CR, complete remission; PR, partial remission; SD, stable disease; PD, progressive disease. [file Image_7.tif]
